# Toll-Like Receptor 2 (TLR2) Plays a Major Role in Innate Resistance in the Lung against Murine Mycoplasma

**DOI:** 10.1371/journal.pone.0010739

**Published:** 2010-05-20

**Authors:** Wees Love, Nicole Dobbs, Leslie Tabor, Jerry W. Simecka

**Affiliations:** Department of Molecular Biology and Immunology, University of North Texas Health Science Center in Fort Worth, Fort Worth, Texas, United States of America; Louisiana State University, United States of America

## Abstract

Mycoplasma lipoproteins are recognized by Toll-like receptors (TLR), but TLRs' role in responses to infection are unknown. *Mycoplasma pulmonis* is a naturally occurring respiratory pathogen in mice. In the current study, we used TLR-transfected HEK cells and TLR2^−/−^ bone marrow-derived dendritic cells to demonstrate TLR2-mediated events are important in the initial host-mycoplasma interactions promoting cytokine responses. As we found alveolar macrophages expressed TLR1, TLR2 and TLR6 mRNAs, a role for TLR2 in innate immune clearance in lungs was examined. Three days post-infection, TLR2^−/−^ mice had higher *M. pulmonis* numbers in lungs, but not in nasal passages. However, TLR2^−/−^ mice had higher lung cytokine levels, indicating TLR2-independent mechanisms are also involved in host responses. Thus, TLR2 plays a critical role in the ability of innate immunity to determine *M. pulmonis* numbers in the lung, and it is likely that early after respiratory infection that TLR2 recognition of *M. pulmonis* triggers initial cytokine responses of host cells.

## Introduction

Mycoplasma infection is a leading cause of pneumonia worldwide. In the United States, *Mycoplasma pneumoniae* accounts for as many as 30% of all cases of pneumonia [1], [2], [3]. Mycoplasmas cause an atypical pneumonia, and in humans, *M. pneumoniae* infection can exacerbate pre-existing respiratory diseases [4], [5], [6]. Mycoplasma infection is also a noted problem in livestock, with a major economic impact worldwide [7]. *M. pulmonis* is a naturally occurring pathogen of rats and mice and the etiological agent responsible for murine respiratory mycoplasmosis (MRM) [7]. MRM causes an atypical pneumonia with both acute and chronic stages to the disease and is an excellent animal model used to gain insight into the pathologies caused by mycoplasma respiratory diseases [8]. As with other mycoplasma diseases, *M. pulmonis* disease has an immunopathologic element to disease progression. In fact, elements of both the innate [9], [10], [11] and adaptive [12], [13], [14] arms of the immune system play a role in the progression and intrapulmonary clearance of the disease. Thus, it is clear that the mechanisms governing the recruitment of inflammatory cells and control of mycoplasma infection will ultimately determine the severity of mycoplasma respiratory disease.

The initial molecular interactions between the invading mycoplasma and the host that play a role in the outcome of an infection remain to be fully determined. Currently, it is believed that attachment to the respiratory epithelium is the first step in colonization of the host, and interactions with the alveolar macrophages (AM) are critical in determining the levels of infection [1]. However, the molecular intermediates that mediate this recognition remain obscure. Toll-like receptors (TLRs) are a highly conserved family of type I transmembrane receptors that recognize specific pathogen-associated molecular patterns (PAMPs), e.g. LPS, lipotechoic acid and other bacterial wall components. The recognition of purified mycoplasma lipoproteins to TLRs is well documented [15], [16], [17]. Specifically, TLR1, TLR2 and TLR6 are implicated in the recognition of mycoplasma lipoproteins stemming from several mycoplasma strains [16], [17], [18], [19]. TLR2 dimerizes with either TLR1 or TLR6 to enhance the recognition of lipoproteins and augment the cellular cytokine response. It is clear that TLRs play a role in the recognition of mycoplasma lipoproteins and could have an impact on host responses. In fact, studies suggest that *M. pneumoniae* may modulate mucin production by airway cells [20], [21]. One study suggests that mycoplasma infection stimulates mucin production in mice or a human epithelial cell line through interaction with TLR2 [20]. The generation of Th2 type responses in a murine asthma model is also associated with down regulation of TLR2 expression and the concomitant decreased in clearance of *M. pneumoniae* [22]. These results are consistent with a role of TLR2 in clearance of mycoplasma, which is supported by studies demonstrating that stimulation of macrophages with TLR agonists can initiate antimicrobial immune responses [23], [24], as well as the production of pro-inflammatory [23], [25], [26] and chemotactic factors [27], [28]. Thus, TLR signaling is likely critical in initiating and/or influencing the host's responses to mycoplasma infection.

The intrapulmonary clearance of *M. pulmonis*, a natural pathogen of mice, is dependent on innate immune mechanisms. There is a body of evidence linking AMs to the intrapulmonary clearance of the organism. AM are able to bind, ingest and kill *M. pulmonis in vitro* and *in vivo* [9], [29], [30]. The intrapulmonary depletion of AMs decreases the resistance to infection of resistant mice strains (e.g. C57BL/6) to levels consistent with susceptible strains (e.g. C3H) [9]. There are likely other mechanisms of innate immunity that contribute to control of infection. Currently, the receptors mediating the recognition of viable *M. pulmonis* by AM and other cells that mediate innate immune mechanisms are unknown. We hypothesize that AM and other innate immune cells recognize viable *M. pulmonis* through TLR2-dependent mechanisms, and that this recognition augments the host's cytokine response and their ability to resist infection. In this study, we investigate the TLR recognition of viable *M. pulmonis* and determine the effects this recognition has on disease pathogenesis. We show that TLR1, TLR2 and TLR6 are utilized in the recognition of viable *M. pulmonis in vitro*. In addition, we show the expression of TLR1, TLR2 and TLR6 in bronchoalveolar lavage (BAL) cells of C57BL/6J mice. Furthermore, TLR2^−/−^ animals show an impaired resistance to infection in the lower respiratory tracts. However, TLR2^−/−^ mice also had higher lung cytokine levels, indicating TLR2-independent mechanisms are also involved in later host responses. Together, our findings show that TLR2 recognizes *M. pulmonis*, and this interaction can promote early cytokine responses and resistance to mycoplasma infection in the respiratory tract.

## Results

### Viable *M. pulmonis* recognition can be mediated by TLR2 and enhanced when TLR1 or TLR6 are co-expressed

As previous studies suggest that mycoplasma lipoproteins are recognized by TLR2 in conjunction with TLR1 and/or TLR6 [18], [19], [31], these receptors may be involved in the recognition of viable mycoplasma by macrophages and other immune cells. Human embryonic kidney (HEK) cells are excellent for TLR-specific mechanistic studies. In the present studies, HEK cell lines, stably transfected to express murine TLRs, were infected with viable *M. pulmonis* to determine whether these TLRs are involved in recognizing viable *M. pulmonis*. TLR-mediate activation of the HEK cells results in the production of IL-8, whose levels were used to monitor the recognition and level of cell stimulation. As a positive control, we stimulated the HEK cell lines with the synthetic diacylated lipoprotein, FSL-1, a known TLR2 agonist. As a control for cell activation independent of TLR activation, we utilized the stock HEK cell line known to have null or low basal expression of all the TLRs, and therefore should produce little if any IL-8 in response to stimulation with TLR agonists. To further confirm the specificity of our cell responses, we also stimulated the cell lines with LPS, a known TLR4/MD2/CD14 agonist.

**Figure 1:**
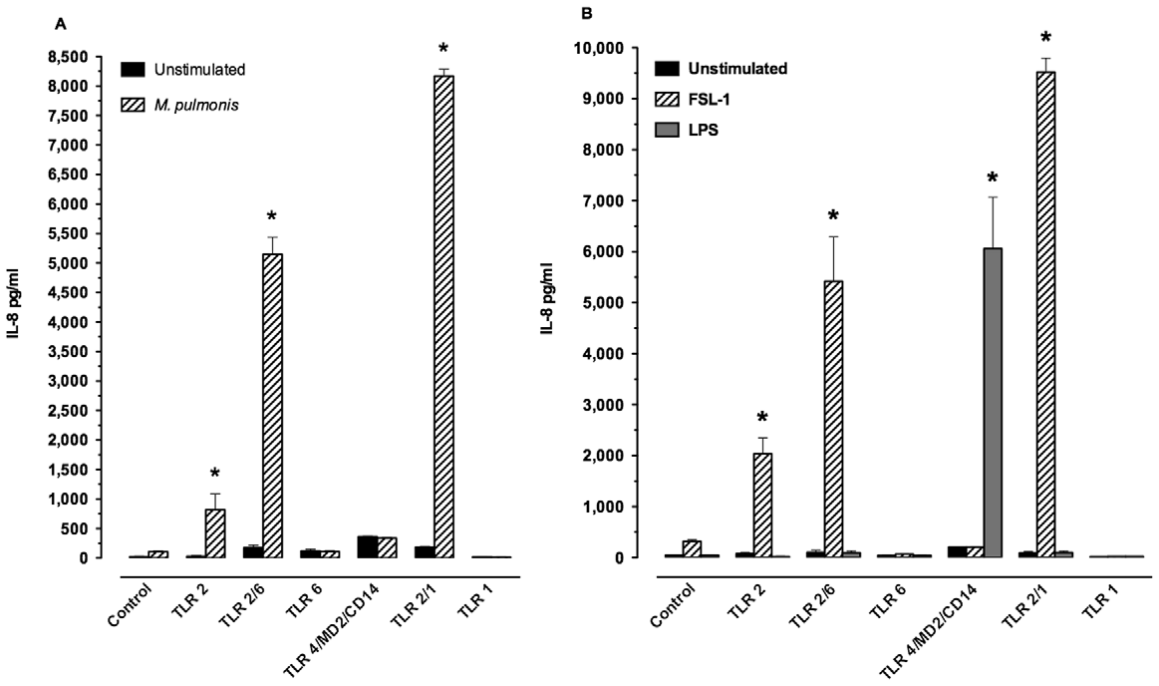
Viable *M. pulmonis* recognition is mediated by TLR2 and enhanced with TLR1 or TLR6 co-expression. HEK cells or those stably transfected to express individual TLRs and their adaptor proteins (TLR2 or TLR4/MD2/CD14) were stimulated for A) 24 hours with viable *M. pulmonis* at 0.7 MOI or B) 6 hours with an *E. coli* derived ultra pure LPS preparation at 10 µg/ml, the TLR2/6 agonist FSL-1 at 1 µg/ml. The supernatants were then collected and assayed for their IL-8 content. Data are expressed mean ± SEM of IL-8 pg/ml production of each agonist. An asterisk “*” denotes a *P* value ≤0.05, as compared to unstimulated cells. This experiment was done three times in triplicate.

Stimulation of the cell lines demonstrated a role for TLR1, 2, and 6 in the recognition of *M. pulmonis*. Maximum stimulation with live *M. pulmonis* organisms occurred at 24 hours (Figure 1A). *M. pulmonis* stimulated the TLR2-expressing cell lines to produce IL-8 (*P*≤0.05), while the TLR4/MD2/CD14-expressing cell line was unresponsive (Figure 1B). To confirm specificity of TLR-mediated responses, HEK cell lines were stimulated with either a TLR4 or TLR2 agonist. Purified TLR agonist controls, LPS and FSL-1, had optimal and maximal stimulation at 6 hours (Figure 1B). As expected, the TLR4/MD2/CD14-expressing cells responded to LPS stimulation (*P*≤0.05) while TLR2-expressing cells did not. Similarly, with FSL-1, a TLR2 agonist, there was an increase in IL-8 production by the TLR2-expressing cell lines with a further enhancement of IL-8 production when either TLR1 or TLR6 was co-expressed with TLR2 (*P*≤0.05, Figure 1). As expected, the TLR4/MD2/CD14-expressing cell line did not respond to FSL-1. These results confirmed the specificity of the responses. Thus, *M. pulmonis* stimulated these cells using TLR2, but not TLR4, to stimulate a cytokine response.

### TLR2 is critical in mycoplasma-induced *in vitro* cytokine responses

The above studies demonstrated that TLR2 can mediate cellular responses to *M. pulmonis* infection, but there is a possibility that other receptors, independent of TLR2, could similarly mediate cytokine responses. To determine if TLR2 is critical in cytokine responses from immune cells, we generated bone marrow derived dendritic cells (BMDC) from TLR2^−/−^ and wild-type C57BL/6J mice. The BMDC were infected with viable *M. pulmonis* for 24 hours. In contrast to HEK cells, murine cells do not produce IL-8. However, IL-6 and TNF-α are produced in response to *M. pulmonis* infection *in vivo* [32], and therefore, the levels of these cytokines were used to measure cell activation, rather than IL-8.

**Figure 2:**
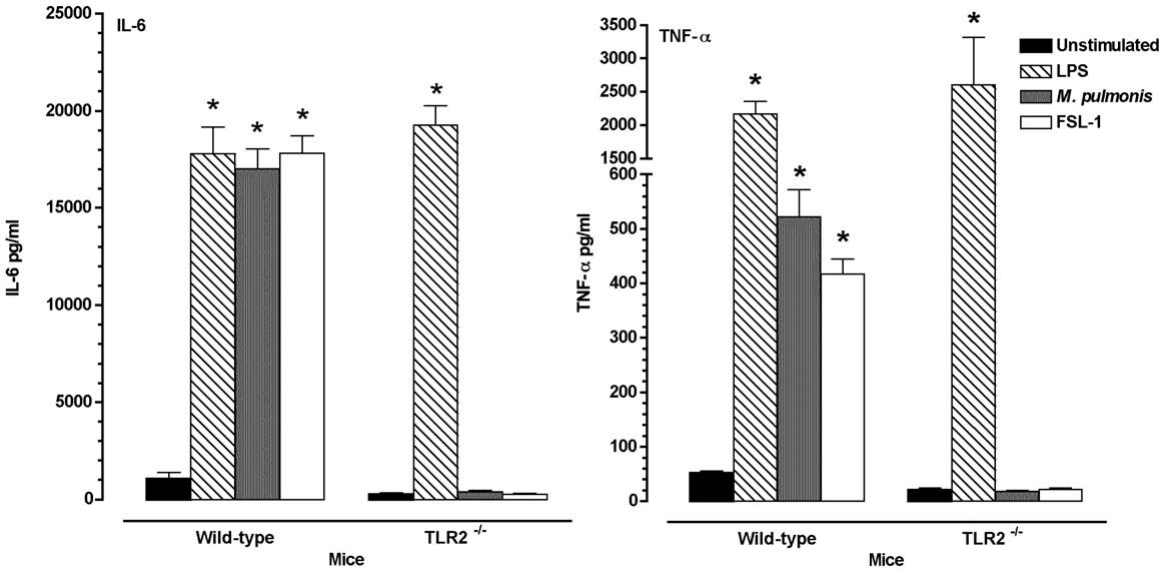
Impaired production of IL-6 and TNF-α by BMDC generated from TLR2^−/−^ mice after mycoplasma stimulation. BMDC were stimulated for 24 hours with an ultra pure LPS at 10 µg/ml or viable *M. pulmonis* at 50 MOI. The supernatants were then collected and assayed for their IL-6 and TNF-α content. Data are expressed mean ± SEM of IL-6 and TNF-α pg/ml production of each agonist. An asterisk “*” denotes a *P* value ≤0.05, as compared to unstimulated cells. This experiment was done twice in triplicate.

Cells from TLR2^−/−^ mice were impaired in their ability to respond to *M. pulmonis* infection. The BMDC from the TLR2^−/−^ mice had no increase in IL-6 and TNF-α production when compared to unstimulated controls, while BMDC from the WT mice had significant (*P*≤0.05) production of both cytokines in response to *M. pulmonis* and the TLR2 agonist, FSL-1 (Figure 2). Both populations of BMDC responded well to LPS, a TLR4 agonist. Thus, TLR2 was found to be critical in the optimal cytokine production by BMDC in response to *M. pulmonis* infection.

### TLR1, TLR2, and TLR6 mRNA are expressed in bronchoalveolar lavage (BAL) cells from C57BL/6J mice

Macrophages are important in the initial response to *M. pulmonis* infection in lungs of mice [9], [29], [30]. To determine if TLR1, TLR2, and TLR6 were expressed in pulmonary macrophages, the BAL cells were collected from uninfected mice and TLR1, TLR2, and TLR6 mRNA levels determined. Previous studies demonstrated that BAL cells are predominately macrophages [9], and we confirmed that cell collected using BAL were primarily alveolar macrophages through their expression of CD11c and F480 [93.4±0.9% CD11c^+^, F480^+^ cells in BAL (Mean ± SEM, n = 9)] [33].

**Figure 3:**
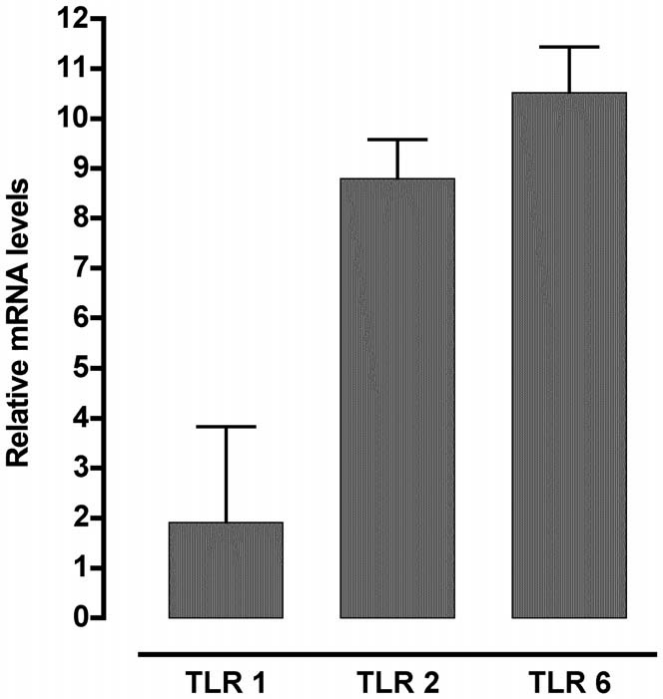
TLR1, TLR2, and TLR6 mRNA are expressed in BAL from mice. The BAL fluid of two mice was collected and pooled. The cells were pelleted, RNA isolated, and cDNA was generated. The primary transcript levels of TLR1, TLR2, and TLR6 were determined using real-time PCR. The data is expressed as the mean ± SEM of the relative levels of mRNA after normalized with GAPDH mRNA expression. This experiment was done a total of three times, and data represented were combined from all three experiments.

TLR mRNA expression of BAL cells was consistent with these receptors being expressed on these cells and having the potential to contribute to the initial responses to mycoplasma species infection in the lung. Specifically, TLR1, TLR2, and TLR6 mRNA were expressed in BAL cells (Fig. 3). TLR1 was expressed at the lowest levels of the three TLRs assayed with TLR6 having the highest expression.

### TLR2^−/−^ mice are impaired in their resistance to *M. pulmonis* pulmonary infection

The above studies demonstrated that TLR2 is involved in generating cytokine responses by immune cells after mycoplasma infection. Base on these results, it is reasonable to suggest that TLR2 recognition is also involved in triggering innate immune mechanisms involved in control of *M. pulmonis* infection. To explore this possibility, *M. pulmonis* numbers were determined in the nasal passages and the lungs in TLR2^−/−^ C57BL/6J and WT mice. C57BL/6 mice were chosen, as they are able to control the levels of *M. pulmonis* pulmonary infection through innate immune mechanisms, primarily alveolar macrophages [9]. Groups of mice were inoculated with *M. pulmonis*, and 72 hours later, *M. pulmonis* numbers in lungs and nasal passages were determined.

**Figure 4:**
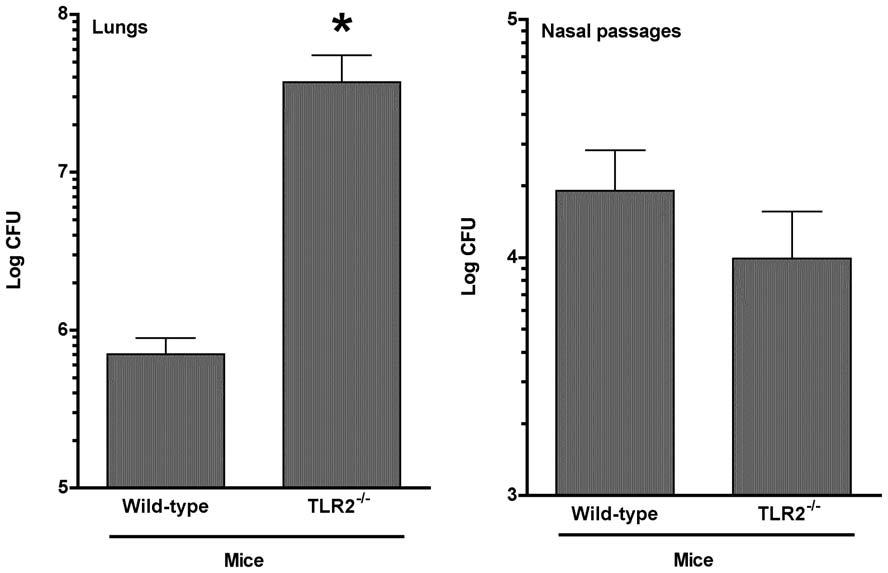
TLR2^−/−^ mice have higher numbers of mycoplasma in the lower respiratory tract following mycoplasma infection. Total CFU numbers were determined in the nasal passages and the lungs in TLR2^−/−^ and wild-type mice. Each mouse was inoculated with *M. pulmonis*. At 72 hours post-infection, the mice were sacrificed, and the lungs and nasal washes were collected from each animal. The experiment was done twice (total of n = 9 animals). An asterisk “*” denotes a *P* value ≤0.05.

**Figure 5:**
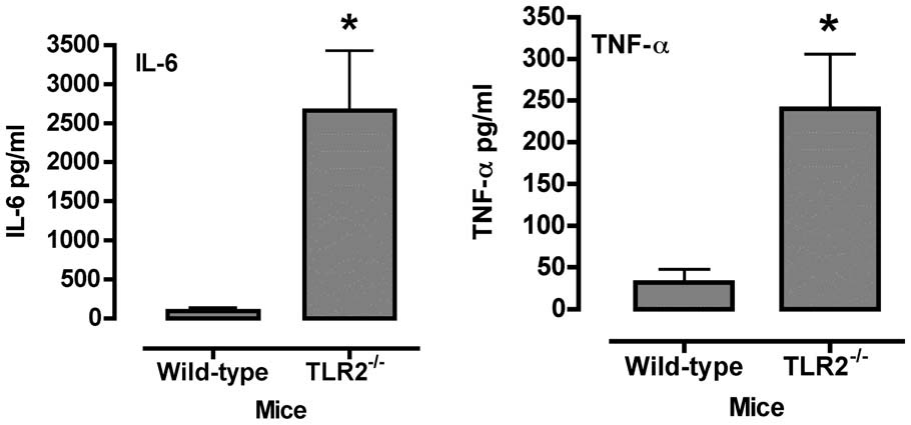
TLR2^−/−^ mice have higher levels of IL-6 and TNF-α in BAL fluids following mycoplasma infection. The levels of IL-6 and TNF-α were determined from lavage fluids collected from the lungs of TLR2^−/−^ and wild-type mice. Each mouse was inoculated with *M. pulmonis*. At 72 hours post-infection, the mice were sacrificed, and three 1 ml BAL were collected from each animal, and cytokine levels in the BAL fluids were determined. The experiment was done twice (total of n = 8 animals). An asterisk “*” denotes a *P* value ≤0.05.

The expression TLR2 expression altered *M. pulmonis* numbers recovered from infected mice. There were no clinical signs of disease or gross inflammatory lesions in TLR2^−/−^ or wild-type C57BL/6J mice by 72 hours post-infection. However, at this time, TLR2^−/−^ mice had approximately 2-log higher numbers of *M. pulmonis* in lungs, while no significant difference was found in the nasal passages (Figure 4). We also found a corresponding increase in IL-6 and TNF-α levels in BAL fluids from TLR2^−/−^ mice (Figure 5). Thus, TLR2^−/−^ mice were impaired in their ability to control *M. pulmonis* infection in the lungs, which likely leads to increased inflammatory responses.

## Discussion

The identification of receptors involved in the recognition of viable *M. pulmonis* by macrophages and other cells is critical in understanding the signals that influence the development of host responses that influence mycoplasma disease pathogenesis. TLR2-mediated recognition of mycoplasma lipoproteins, derived from several mycoplasma strains, is well documented in the literature [15], [16], [18], [19], [34], [35], but there are little or no studies demonstrating the ability of TLR2 to recognize infecting organisms or determining the outcome of this interaction during infections. In this study, we demonstrate that cellular recognition of viable *M. pulmonis* is mediated by TLR2. In support, HEK cells expressing TLR2 responded to *M. pulmonis* infection, and TLR2^−/−^ dendritic cells did not respond to infection whereas wild type cells produced significant cytokine responses. Although there still might be other TLR2-independent mechanisms involved in the recognition of *M. pulmonis*, the lack of responses by TLR2^−/−^ cells indicates that TLR2 plays a critical role in the initial recognition of *M. pulmonis*. In addition, the responses mediated by TLR2 on HEK cells were significantly enhanced with the co-expression of TLR1 or TLR6. This is consistent with previous studies demonstrating that TLR2, in concert with TLR1 and/or TLR6, is involved in the recognition of purified lipoproteins derived from several other mycoplasma strains [15], [16], [18], [19], [34], [35]. The current paradigm suggests that TLR2 dimerizes with TLR1 to recognize triacylated lipoproteins [36] and with TLR6 to recognize diacylated lipoproteins [37], [38]. However, recent reports suggest that the discrimination in recognition of bacterial lipoproteins might be more complex [37]. In fact, a dipalmityolated lipoprotein from *M. pneumoniae* was shown to activate NF-κβ through TLR2, TLR6, and TLR1 [16]. To our knowledge, these are the first studies that demonstrate the TLR recognition of infection by *M. pulmonis*. Thus, these studies demonstrate *M. pulmonis* recognition is mediated by TLR2 and suggests that *M. pulmonis* expresses both di- and triacylated lipoproteins on its surface, which mediate these responses.

TLR2 recognition of viable mycoplasma species leads to stimulation of innate immune mechanisms controlling the level of infection in lungs, but these receptors have no apparent effect in nasal passages. Similarly, other bacterial pathogens, such as *Borrelia burgdorferi* [39], *Streptococcus pneumoniae* [40], [41] and *Mycobacteria* BCG [42], are recognized by TLR2, resulting in clearance of infection in lungs. In *M. pulmonis* infections, the TLR2^−/−^ mice had 2-log higher *M. pulmonis* numbers in the lungs when compared to their WT counterparts, but there was no difference in nasal passages. In lungs, clearance of organisms is mediated by innate immunity, specifically alveolar macrophages [30]. C57BL/6 strain of mice is resistant to *M. pulmonis* pulmonary infection due to the effectiveness of their alveolar macrophages. We found that indeed TLR1, TLR2, and TLR6 mRNA's were expressed by macrophage-enriched BAL cells of C57BL/6J mice, supporting TLR2, along with TLR1 and/or TLR6, as a receptor contributing to the killing of *M. pulmonis*. In support, TLR activation is linked to the enhanced phagocytosis of other microbes [43]. Interestingly, TLR1 mRNA was expressed at the lowest levels, suggesting TLR6 may play the bigger role in TLR2-mediated responses by BAL cells. Further studies are needed to assess whether differences in TLR expression occur after infection and whether expression differs between tissues, such as macrophages and epithelial cells, which could result in differential sensitivity to infection between cell types. Thus, TLR2 recognition of mycoplasma respiratory infections by alveolar macrophages and other cells conferring innate immunity leads to clearance and/or control of these organisms in the lungs, but does not have a similar effect in nasal passages in this same period.

Based on our *in vitro* studies, we predicted that cytokine production would be reduced in infected mice; however, TLR2^−/−^ mice had higher levels of cytokines in BAL fluids 72 hours after infection, corresponding to the higher numbers of *M. pulmonis* in the lung. Association of higher *M. pulmonis* numbers and inflammatory cytokine levels is consistent with previous studies [32], [44]. There are clearly additional TLR2-independent mechanisms in the lung, perhaps initiated by cell damage due to higher numbers of organisms, contributing to host responses to infection. However, TLR2 recognition of viable *M. pulmonis* is most likely still important in the host's responses early after respiratory infection. In fact, previous studies indicate that modulation of TLR2 expression on respiratory epithelium can alter mucin production in response to infection with the human pathogen, *M. pneumoniae* [20], [22]. In support, ongoing studies (unpublished) in our laboratory demonstrate that TLR2 is involved in stimulation of cytokine responses by respiratory epithelial by either *M. pulmonis* or *M. pneumoniae*. Thus, both TLR2-dependent and –independent responses are elicited after mycoplasma infection, and most likely each contribute to the progression of infection and disease.

In summary, it is clear that TLR2-mediated events are important in the initial interactions between the host and mycoplasma. Using a naturally occurring murine model of mycoplasma respiratory disease, our results demonstrate that *M. pulmonis* interactions with TLR2 play as major role in host recognition and innate immunity's ability to control of the level of pulmonary infection. In addition, dimers of TLR2 with TLR1 or TLR6 are involved in the recognition on viable *M. pulmonis* by host cells, in addition to macrophages, leading to stimulation of cells and cytokine/chemokine production. As lipoproteins from other mycoplasmas can stimulate cells using TLR2 [15], [16], [17], our studies support a role of TLR2, along with TLR1 and/or TLR6, recognition in infection and disease pathogenesis in other mycoplasma infections, including those that affect humans. As a supporting example, previous studies [20], [22] indicate that TLR2 expression may be linked to mucin production by epithelial cells and clearance of the human pathogen, *M. pneumoniae*, in a mouse infection. Another intriguing possibility is that TLR2-mycoplasma interactions on antigen presenting cells, such as dendritic cells and macrophages, and other immune cells may also modulate the types of adaptive immune responses generated in response to infection. Studies in other systems support this possibility [45], [46], [47]; in fact, IFN-γ production in response to *M. pneumoniae* infection of mice may in part depend upon TLR2 expression [21]. Future studies are needed to determine if TLR2, TLR1, and/or TLR6 recognition of *M. pulmonis* are involved in adaptive, as well as innate, host responses. If so, this may be harnessed in developing new immunotherapies, including enhancing innate resistance and optimizing vaccine strategies.

## Materials and Methods

### Ethics statement

All animals were handled in strict accordance with good animal practice as defined by the relevant national and/or local animal welfare bodies, and all animal studies were approved by University of North Texas Health Science Center Institutional Animal Care and Use Committee (IACUC).

### Mice

C57BL/6J (wild-type, WT) and B6.129S1-*TLR2^tm1Kir^*/J (TLR2^−/−^) mice, tested to be viral and mycoplasma free, were obtained from The Jackson Laboratory (Bar Harbor, MN). Mice were housed in sterile microisolator cages supplied with sterile bedding, food and water given *ad libitum*. Mice used in the studies were between 9–15 weeks of age. Female mice were used in all studies. Before experimental infection, mice were anesthetized with an intramuscular injection of ketamine/xylazine.

### Cell lines

Human embryonic kidney 293 (HEK) cell lines were obtained from InvivoGen, (San Diego, CA), and included HEK (control) and HEK cells stably transfected to express murine TLR1, TLR2, TLR6, TLR2/6, TLR2/1 or TLR4/MD2/CD14. All cells were cultured in DMEM high glucose containing 10% FBS and 2 mM L-glutamine without the use of antibiotics, and the HEK cell lines were used prior to 10 passages.

### Mycoplasma

The UAB CT strain of *M. pulmonis* was used in all experiments. Stock cultures were grown, as previously described [48], for *in vitro* stimulation studies 1 ml aliquots frozen at −80°C were thawed and grown in 9 ml Hayflick's medium for 3 hours at 200 rpm. The resulting growth was spun down at 10,000 rpm for 20 minutes, washed in serum free Dulbecco's Modified Eagle Medium (DMEM) high glucose (Hyclone, Logan, UT) and reconstituted in a total volume of 5-ml of serum free DMEM. For infections, mice were anesthetized with an intraperitoneal injection of ketamine/xyalzine, and then they received an intranasal inoculum of 20 µl containing 2×10^6^ cfu of *M. pulmonis* strain CT [48].

### Generation of bone marrow derived dendritic cells (BMDC)

Bone marrow derived dendritic cells were generated as previously described [49]. Femora and tibia were collected from mice, and the bones were suspended in wash medium containing RPMI 1640 (Hyclone), 10% FBS (Hyclone), antibiotic/antimycotic solution (Life Technologies, Grand Island, NY), and HEPES buffer (Fisher Scientific, Pittsburgh, PA). The bones were twice washed in medium and also soaked in 70% ethanol between washes. The epiphyses were cut, and the marrow flushed from the bones with wash medium. Epiphyses were finely minced and added with the flushed marrow. The suspension was passed through a 250 µm nylon mesh to remove any unwanted debris. The cell suspension was then spun down, and red cells removed using ACK lysis buffer [50]. The cells were then spun down, re-suspended in wash medium and counted. Once counted, the cells were placed in cell culture flasks at a concentration of 10^7^ cells/ml. The cells were cultured in the presence of IL-4 (20 ng/ml, Invitrogen) and GM-CSF (20 ng/ml, Invitrogen). Culture medium was changed every two days, and the cells were harvested on day 6.

### *In vitro* cell stimulations

The HEK stable transfectants (InvivoGen) were seeded at 2×10^5^ cells/well in 24-well tissue culture treated plates. The cells were washed and seeded in serum-free DMEM high glucose cell culture medium (Hyclone). The agonists, FSL-1 (1µg/ml, InvivoGen), Ultrapure LPS, *E. coli* 0111:B4 (10 µg/ml, InvivoGen), and whole organism, *M. pulmonis* [multiplicity of infection (MOI) of 0.7 cfu/cell], were suspended in serum-free DMEM high glucose (Hyclone) and added to the wells for a total volume of 500 µl/well. The supernatants were collected at 6- and 24-hour time points. BMDC were similarly stimulated with Ultrapure LPS or *M. pulmonis* (MOI of 50), and cell culture supernatants were collected at 24 hours after stimulation. Culture supernatants were stored at −80°C until assayed.

### Bronchoalveolar lavage (BAL) cells and fluid

The BAL was performed as previously described [51]. Mice were killed by lethal injection, and their tracheas were exposed and ligated distal to the larynx. A sterile 22G ProtectIV Plus catheter (MEDEX, Carlsbad, CA) was inserted approximately 2 to 3 mm into the lumen, and the lungs were then lavaged 3 times with 1 ml aliquots of PBS solution (Hyclone). The lavages were pooled, and cells pelleted using centrifugation at 200×g for 10 min at 4°C. The BAL fluids were aliquoted and stored at −80°C, and cell pellets were resuspended in 500 µl of Trizol (Invitrogen Life Technologies, Carlsbad CA).

Alveolar macrophages express CD11c in addition to other macrophage markers, such as F480 [33]. BAL cells were suspended in 50 µl of staining buffer (1× PBS, 2mM EDTA, 2% FBS) with diluted antibodies PE anti-mouse CD11c (BD Pharmingen, catalog # 553802), APC anti-mouse F4/80 (Caltag Laboratories, catalog # MF48005) and purified anti-mouse CD16/CD32 Fc Block (BD Pharmingen, catalog # 553142). Cells were incubated with antibodies for 30 min at 4°C, and then quenched with 1 mL of staining buffer and centrifuged (just as above). Nine samples were stained and 2 samples were unstained (except for the FcBlock). Samples were resuspended in 300 µl of staining buffer and run on a BD LSRII Flow Cytometer using BD FACSDiva Software v6. The samples were analyzed using FlowJo v9.0.1 (TreeStar). Stained samples were compared to unstained samples and alveolar macrophages were identified as CD11c^+^ (PE) and F4/80^+^(APC).

### RNA extraction

RNA from BAL cells was isolated using Trizol RNA isolation reagent, as recommended by the manufacturer (Invitrogen) [44]. 500 µl of TRIZOL were added to the pellet and pipetted to ensure lysis of the sample. The homogenates were frozen at −80°C until needed. Chloroform was added to the thawed homogenates and centrifuged at 12,000×*g* (4°C) for 30 min. RNA was precipitated by adding isopropanol to the aqueous phase and centrifuging samples at 12,000×*g* (4°C) for 10 min. The RNA pellet from each sample was washed twice with 75% ethanol by vortexing and subsequent centrifugation for 5 min at 7,500×*g*, and then re-suspended in diethylpyrocarbonate-treated water. The samples were stored at −80°C until further use.

### Cytokine ELISA

Human IL-8 was measured using OptEIA IL-8 ELISA set (BD Pharmingen). BAL fluid and supernatant from BMDC stimulation experiments were evaluated for the cytokine expression of IL-6 and TNF-α using a mouse cytokine Milliplex™ Map kit (Millipore, Billerica, MA). Cytokine assays were run as manufacturer instructed. Samples were read using a Bio-Plex 100 system (Bio-Rad, Hercules, CA). Cytokine levels were determined by comparison with standard curves generated from murine recombinant cytokines and analyzed using Bio-Plex Manager 4.0 software (Bio-Rad).

### Toll-Like receptor mRNA detection by real time-PCR

cDNA was synthesized from total RNA samples in a 100-µl reaction using the Taqman Gold RT-PCR kit (Applied Biosystems, Foster City, CA) according to the manufacturer's instructions. Sybr-green real-time PCR was performed using RT^2^ SYBR Green/ROX qPCR Master Mix and primers for TLR1, TLR2, TLR6 and the housekeeping gene GAPDH (SuperArray Bioscience Corporation, Frederick, MD). Real-time PCR was performed in 25 µl SmartCycler tubes (Cepheid, Sunnyvale, CA), and the real-time PCR products were amplified using a SmartCycler system (Cepheid) at 95.0°C for 10 min, followed by 40 cycles of 95.0°C for 15 s and 60.0°C for 60 s. The threshold of the growth curve (*C_T_*) was set at a value of 30 using the SmartCycler software. The expression of the housekeeping gene, GAPDH, was used to normalize the data. The formula for the normalization (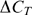) between the amplified TLR gene and the normalizer (GAPDH) is
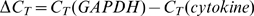



### Determination of mycoplasma numbers

Following infection, lungs and nasal passages were quantitatively cultured as described elsewhere [48]. Lungs were minced, and both lungs and nasal passage washes were placed in mycoplasma broth medium. The samples were sonicated for 45–60 seconds, and 1∶10 serial dilutions were prepared. 20 µl of each dilution were plated onto mycoplasma agar medium. After 7 days of incubation at 37°C, cfu were counted.

### Statistical Analyses

Data results were analyzed using two-way ANOVA followed by multigroup comparisons or Students' t-test when appropriate. When necessary, the data was logarithmically transformed prior to analysis. The data were analyzed using the Instat and Prism software programs (GraphPad, San Diego, CA). A *P* value ≤0.05 was considered statistically significant.
